# Annealing temperature effects on photoelectrochemical performance of bismuth vanadate thin film photoelectrodes†

**DOI:** 10.1039/c8ra04887h

**Published:** 2018-08-16

**Authors:** Le Shi, Sifei Zhuo, Mutalifu Abulikemu, Gangaiah Mettela, Thangavelu Palaniselvam, Shahid Rasul, Bo Tang, Buyi Yan, Navid B. Saleh, Peng Wang

**Affiliations:** Water Desalination and Reuse Center, Division of Biological and Environmental Sciences and Engineering, King Abdullah University of Science and Technology Thuwal 23955-6900 Saudi Arabia peng.wang@kaust.edu.sa; Division of Physical Science and Engineering, King Abdullah University of Science and Technology Thuwal 23955-6900 Saudi Arabia; Department of Civil, Architectural and Environmental Engineering, University of Texas at Austin Austin TX 78712 USA

## Abstract

The effects of annealing treatment between 400 °C and 540 °C on crystallization behavior, grain size, electrochemical (EC) and photoelectrochemical (PEC) oxygen evolution reaction (OER) performances of bismuth vanadate (BiVO_4_) thin films are investigated in this work. The results show that higher temperature leads to larger grain size, improved crystallinity, and better crystal orientation for the BiVO_4_ thin film electrodes. Under air-mass 1.5 global (AM 1.5) solar light illumination, the BiVO_4_ thin film prepared at a higher annealing temperature (500–540 °C) shows better PEC OER performance. Also, the OER photocurrent density increased from 0.25 mA cm^−2^ to 1.27 mA cm^−2^ and that of the oxidation of sulfite, a hole scavenger, increased from 1.39 to 2.53 mA cm^−2^ for the samples prepared from 400 °C to 540 °C. Open-circuit photovoltage decay (OCPVD) measurement indicates that BiVO_4_ samples prepared at the higher annealing temperature have less charge recombination and longer electron lifetime. However, the BiVO_4_ samples prepared at lower annealing temperature have better EC performance in the absence of light illumination and more electrochemically active surface sites, which are negatively related to electrochemical double-layer capacitance (*C*_dl_). *C*_dl_ was 0.0074 mF cm^−2^ at 400 °C and it decreased to 0.0006 mF cm^−2^ at 540 °C. The OER and sulfide oxidation are carefully compared and these show that the efficiency of charge transport in the bulk (*η*_bulk_) and on the surface (*η*_surface_) of the BiVO_4_ thin film electrode are improved with the increase in the annealing temperature. The mechanism behind the light-condition-dependent role of the annealing treatment is also discussed.

## Abstract 2

The effects of annealing treatment on crystallization behavior, grain size, electrochemical (EC) and photoelectrochemical (PEC) oxygen evolution reaction (OER) performances of bismuth vanadate (BiVO_4_) thin films are investigated in this work.

## Graphical abstract

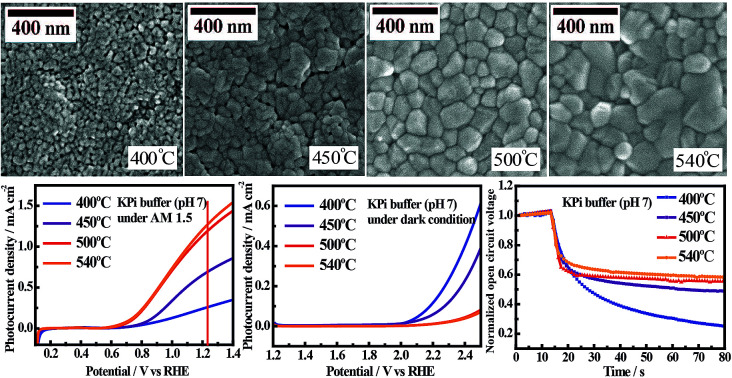

## 1. Introduction

Harvesting solar energy is an effective alternative energy pathway and is desired to mitigate environmental impacts from fossil fuel based energy production.^1,2^ Photoelectrochemical (PEC) water splitting with metal oxide semiconductors has attracted enormous attention in solar energy conversion.^3–5^ Recently, bismuth vanadate (BiVO_4_), an n-type bimetallic oxide semiconductor with a narrow band gap (∼2.4 eV), suitable band position, and relatively high stability against photo-corrosion, has distinguished itself as a promising photoanode-material.^6,7^ However, pristine BiVO_4_ has poor electron–hole separation^8^ and exhibits slow water oxidation kinetics,^9^ which are major obstacles to utilization of the photoreactive capacity of this material.

Recent research has invested into multiple strategies in improving the PEC performance of BiVO_4_, including heterojunction formation, (*e.g.*, Co_3_O_4_/BiVO_4_,^10^ SnO_2_/BiVO_4_,^11^ WO_3_/BiVO_4_,^12^ TiO_2_/BiVO_4_,^13,14^ AgFeS_2_–BiVO_4_ (ref. 15)), doping with Mo or W,^16–18^ addition of electron collectors,^19^ Plasmonic enhancement,^20–23^ and addition of co-catalysts, such as Co–Pi,^9,24^ α-Fe_2_O_3_,^25^ NiCoO_2_,^26^ and Ir–COOH.^27^ A closer investigation reveals that there exists a large variation in the generated current (Table S1†). Literature reports also highlight the lack of consistency in sample preparation process, including the precursor composition, annealing temperature, *etc.* (Table S1†). Different materials need different optimal annealing temperature and different synthesis conditions. In fact, the annealing temperature is recognized as an essential and critical step in crystallization of BiVO_4_,^28–31^ but how annealing temperature affects the PEC performance, specially that of BiVO_4_ thin film electrode, is largely unexplored.

In this study, we systematically investigate the effects of annealing temperature (400–540 °C) on crystallization behavior, surface morphology, and grain size of BiVO_4_ thin film photoelectrodes, prepared by spin-coating. The electrochemical (EC) and PEC performances of the BiVO_4_ thin film photoelectrodes are carefully assessed in relation to the annealing temperature and other parameters affected by the annealing temperature. First, under dark condition, BiVO_4_ thin film electrodes prepared at lower temperature produce better electrochemical performance, which might be due to the more electrochemically active sites. Second, under AM 1.5 irradiated conditions, BiVO_4_ thin film electrodes prepared at higher annealing temperature produce higher photocurrent density. The OER photocurrent density was increased from 0.25 mA cm^−2^ to 1.27 mA cm^−2^ for the samples prepared from 400 °C to 540 °C, which might be due to the less charge recombination controlled by higher degree of crystallinity and bigger grain size, and also better crystal orientation for O_2_ evolution. This work will attract attention on the annealing temperature effect on the EC and PEC performance of BiVO_4_ based thin film electrode.

## 2. Experimental

### 2.1 Reagents and materials

Bismuth(iii) nitrate pentahydrate (Bi(NO_3_)_3_·5H_2_O, purity ≥ 98%), vanadyl acetylacetonate (VO(acac)_2_, purity ≥ 98%), acetylacetone (purity ≥ 99%), and methylene blue were procured from Sigma Aldrich (St. Louis, MO, USA). The conductive fluorine-doped tin oxide (FTO) coated glass with a sheet resistance of 7 Ω sq^−1^ was purchased from Xinyan (Xinyan Technology Co., China). All aqueous solutions were prepared with Millipore deionized (DI) water with a resistivity of 18.2 MΩ cm.

### 2.2 Preparation of BiVO_4_ thin film photoelectrodes

The BiVO_4_ thin films were prepared by spin-coating and in a layer-by-layer fashion. First, the precursor solution was prepared by dissolving 0.40 g of Bi(NO_3_)_3_·5H_2_O and 0.22 g of VO(acac)_2_ in 10 mL acetylacetone, under sonication for 10 h with gentle heating at 40 °C; a dark brown and transparent solution of 80 mM of bismuth and 80 mM of vanadium (atomic ratio Bi/V = 1) in acetylacetone was finally obtained.

The fluorine-doped tin oxide glass (FTO glass) was pre-cleaned following a multi-step procedure described as follows: sonication for 20 min in DI water containing 3 wt% sodium dodecyl sulfate (SDS), sonication for another 20 min in DI water, followed by sonication for 20 min in acetone, and finally sonication for 20 min in ethanol (96%) prior to storing in ethanol. The pre-cleaned FTO glass was dried under nitrogen, followed by cleaning with a Plasma cleaner (Harrick Plasma, Ithaca NY, USA) for 2 min, immediately prior to using it as a substrate in spin-coating of the BiVO_4_ thin film.

The precursor solution of 80 μL was spin-coated at 1400 rpm for 20 s on a CHEMAT Technology Spin coater (KW-4A, Northridge, CA) onto a 2.5 × 2.5 cm FTO substrate to ensure complete coverage. Upon evaporation of excess solvent on a hotplate at 100 °C for 2 min in ambient air, the coated FTO was heated up to 400–540 °C for 5 min in air for pre-crystallization. Four pre-crystallization temperatures, *i.e.*, 400, 450, 500 and 540 °C, were investigated in this step. The samples were then cooled for 5 min in ambient condition. Multiple samples with 2, 4, 6, and 8 layers of coatings, were prepared for comparison. After deposition of the final layer, the BiVO_4_ coated FTO electrode was heated on a hotplate at 100 °C for 2 min, followed by annealing in a muffle furnace at the pre-crystallization temperature for 0.6 h and at a heating rate of 2 °C min^−1^ for further crystallization. The samples were labeled as BiVO_4_-*n*, with *n* being the pre-crystallization and annealing temperature.

### 2.3 Material characterization

The surface characteristics of the BiVO_4_-*n* electrodes were investigated by field-emission scanning electron microscopy (FESEM, Zeiss Merlin, Germany). Surface roughness analysis was carried out by atomic force microscopy (AFM, Agilent 5400SPM, Agilent Technologies, USA), at scan areas of 2.0 × 2.0 μm and 5.0 × 5.0 μm for all samples. Powdered X-ray diffraction (XRD, Bruker D8 Advance diffractometer, USA, using Cu Kα radiation, *λ* = 1.5418 Å) was used to characterize the crystallinity of the samples. All samples were analyzed by XRD in the range of 15–70° at a scan rate of 0.5° min^−1^; the crystal sizes of the BiVO_4_-*n* were estimated with the Scherrer formula.^32^ The diffuse reflectance UV-Vis absorption spectra of the samples were recorded with a spectrophotometer (UV 2550, Shimadzu, Japan), with fine BaSO_4_ powder as a reference. In thermal gravimetric analysis (TGA, NETZSCH, Germany),100 μL precursor was vacuum-dried for 5 h prior to the measurements. X-ray photoelectron spectroscopy (XPS) results were collected by an Axis Ultra instrument (Kratos Analytical) under ultrahigh vacuum (<10^−8^ torr) and by using a monochromatic Al Kα X-ray source. The adventitious carbon 1s peak was calibrated at 285 eV and used as an internal standard to compensate for any charging effects.

### 2.4 PEC performance assessment

The PEC performances of the prepared BiVO_4_-*n* photoelectrodes were evaluated by using a three-electrode configuration (Autolab, PGSTAT302N, Metrohm, The Netherlands), where BiVO_4_-*n* electrode was the working electrode, Pt mesh was the counter electrode, and Ag/AgCl in 3 M KCl was the reference electrode. All the three electrodes were immersed in a 60 mL quartz container containing a supporting electrolyte. The supporting electrolyte used in all the PEC systems was 0.1 M phosphate (KPi) buffer solution (pH 7), unless otherwise specified. Prior to PEC measurement, the electrolyte was purged with N_2_ for 30 min to remove dissolved O_2_ from the electrolyte solution. Light was irradiated from a solar simulator (Oriel® Sol1A Class ABB Solar simulators, Newport, USA). The intensity of the light source was calibrated with a Si diode (Model 818, Newport, USA) to simulate air-mass 1.5 global (AM 1.5) solar light illumination (100 mW cm^−2^). Electrochemical impedance spectroscopy (EIS) was carried out by applying an AC voltage amplitude of 10 mV within the frequency range from 10^5^ to 10^−3^ in 0.1 M KPi buffer solution under both dark condition and AM 1.5 illumination. The open-circuit photovoltage decay (OCPVD) was monitored by measuring the photovoltage of samples at open circuit condition under irradiated condition followed by dark condition (to observe decay curves). The incident-photon to current efficiency (IPCE) spectra was acquired under irradiated condition. The light from a 300 W Xe lamp (Model 73404, Newport, USA) was focused through the monochromator (Model: 74125, Newport, USA) onto the working electrode, and the monochromator was incremented in the spectral range (350–600 nm) with a sampling interval of 10 nm and a current sampling time of 50 s. The light intensity and the generated photocurrent were measured with Newport power meter (Model 1918-R, Newport, USA) and Autolab electrochemical workstation (Autolab, PGSTAT302N, Metrohm, The Netherlands), respectively.

## 3. Results and discussions

### 3.1 Preparation of the photoelectrodes

The BiVO_4_ thin film photoelectrodes are prepared by the layer-by-layer spin-coating method. The detailed sample synthesis is described in the Experimental part. It is noteworthy that a gentle heating is necessary for preparing the precursor solution to make sure its coloration changes from green to dark brown before applying it to spin-coating (Fig. S1†).^33,34^ The spin-coating cycles of 2, 4, 6, and 8 are tested and compared in this study. The UV-Vis spectra of the BiVO_4_ samples indicate that the light absorption by the samples increases with the number of layers and begins to plateau at and beyond 6 layers (Fig. S2†). Thus, the BiVO_4_ samples with 6 layers are utilized hereinafter, unless otherwise specified. Using the cross-section SEM image (Fig. S3†), the thickness of the 6-layers BiVO_4_ thin film is estimated to be ∼93 nm, which is near the estimated optimal hole diffusion length in BiVO_4_ (∼100 nm).^34^

There are two heating steps in the sample preparation: brief and multiple heating in ambient air for pre-crystallization upon coating each layer and annealing in a muffle furnace at the end after the completion of coating all layers. To assess the effect of temperature on grain size of BiVO_4_, 400, 450, 500 and 540 °C conditions are investigated in both pre-crystallization and annealing steps. For the purpose of simplification, the temperatures of the pre-crystallization and annealing for the same sample are kept the same in this study. The temperature range is chosen based on the thermal gravimetric analysis (TGA) (Fig. S4†). The TGA result shows a clear weight change of the BiVO_4_ before 400 °C, indicating the conversion of the precursor to BiVO_4_ has to be accomplished above 400 °C, and this is in agreement with the literature report.^35^ Considering the fact that the FTO glass (7 Ω sq^−1^, Xinyan, Inc.) is produced with the annealing temperature of 600 °C, 540 °C is chosen to be the highest annealing temperature in this work. XRD results of BVO-540 samples annealed at different duration time are presented Fig. S5,† which indicate that 0.6 h annealing duration is long enough for complete crystallization.

### 3.2 Crystallinity and light absorption properties of the photoelectrodes

**Fig. 1:**
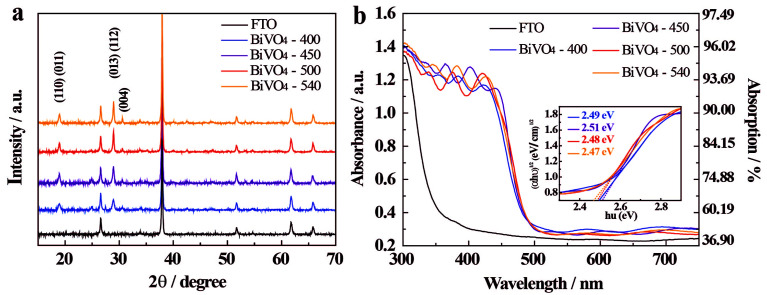
(a) XRD spectra and (b) UV-Vis absorption spectra of BiVO_4_-*n* samples; FTO-glass control spectrum is also presented. Absorption *Y* and absorbance log_10_(1/(1 − *Y*)) were measured between 300 and 750 nm. Inset in (b) shows transformed Kubelka–Munk functions^36^ for each sample as a function of energy of illumination.

The diffraction peaks at 18.9°, 28.9°, 30.56°, 40.05° and 42.5° (Fig. 1a) of the XRD spectrum identify that all BiVO_4_ samples possess a scheelite-monoclinic (s-m) phase (JCPDS 14-0688), which is known for its improved photocatalytic activity in O_2_ evolution among all the BiVO_4_ crystalline phases;^28,37,38^ scheelite-tetragonal (s-t) and zircon-tetragonal (z-t) are the other two crystal phases. It should be noted that no bismuth or vanadium oxide phase impurities have been identified. The additional diffraction peaks observed can be assigned to the FTO substrate, as expected from a thin BiVO_4_ coated glass surface.

The full width at half maximum (FWHM) at the peak (013) (112) is 0.408, 0.312, 0.229, and 0.114 for BiVO_4_-400, BiVO_4_-450, BiVO_4_-500, and BiVO_4_-540, respectively, and this decreasing trend with the increase in annealing temperature indicates that the crystallinity of BiVO_4_ improves with annealing temperature. The crystal sizes are calculated using the Scherrer formula:1
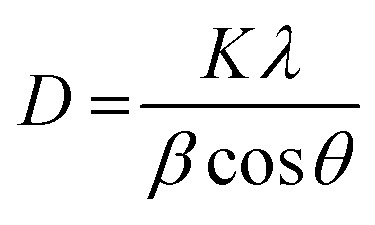
where, *D* is the approximate size of the ordered (crystalline) domains, which may be smaller or equal to the grain size; *K* is a dimensionless shape factor, with a typical value of 0.94 was used in this work; *λ* is the wavelength of the X-ray radiation (1.5418 Å); *β* is the peak width at the half-maximum intensity, after subtracting the instrumental line broadening; *θ* is the Bragg diffraction angle. The crystal sizes calculated based on the (013) (112) peak are listed in Table S2,† which confirms increasing crystal size with annealing temperature.

Moreover, the peak intensity ratios of the (004) and (112) facets are 0.213, 0.172, 0.178, and 0.227 for BiVO_4_-400, BiVO_4_-450, BiVO_4_-500, and BiVO_4_-540, respectively; while those of the (004) and (110) facets are 0.357, 0.313, 0.400, and 0.550 for BiVO_4_-400, BiVO_4_-450, BiVO_4_-500, and BiVO_4_-540, respectively. It is evident that when the annealing temperature is higher than 450 °C, the fraction of exposed (004) surface in BiVO_4_ thin film electrode increases significantly. This enhancement in the exposure of (004) crystal facets is known to be beneficial for PEC water oxidation as these facets may provide active sites for O_2_ evolution.^39–41^ These results demonstrate that the annealing temperature significantly affects the crystallinity and crystal orientation of the BiVO_4_ thin-film. In addition, the diffraction peat at 25° is coming from adsorbed carbon contaminants in the sample preparation process, which can be completely removed by increasing the annealing temperature. XPS analysis was carried out to gain addition insights (Fig. S6†) and all the discussion in details are presented in ESI.†

The effect of the annealing temperature on the light absorption properties is investigated by UV-Vis absorption and diffuse reflectance spectra^7,42,43^ (Fig. 1b and S7†) and the results show no obvious effect in the temperature range tested. This is likely due to the similar thickness of the samples as these are coated with the same number of layers. Interestingly, both periodic absorption and reflection peaks are observed, which are likely a result of a periodically ordered surface morphology and of uniform size of the grains in the BiVO_4_ thin films.^44^

### 3.3 Surface morphology of the photoelectrodes

**Fig. 2:**
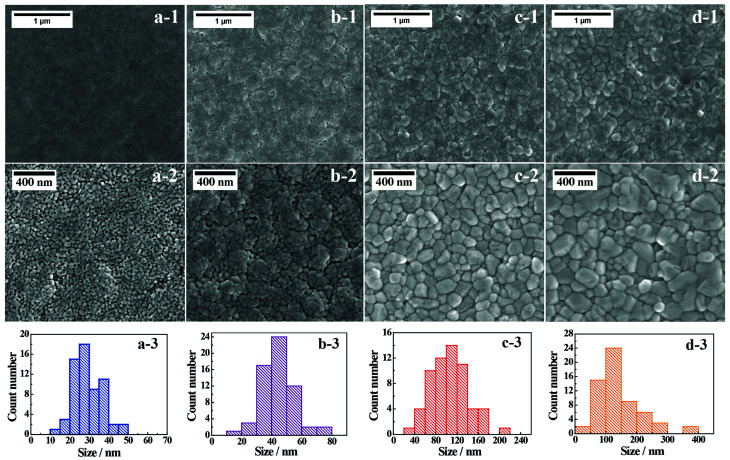
Top view SEM images of (a1 and a2) BiVO_4_-400, (b1 and b2) BiVO_4_-450, (c1 and c2) BiVO_4_-500, and (d1 and d2) BiVO_4_-540. Grain size (nm) distributions derived from SEM images of each sample (a3–d3).

Fig. 2 presents representative SEM images of the BiVO_4_-*n* samples; the crystals appear to be sintered together to form a relatively compact thin film. However, some surface cavities are visible in BiVO_4_-400, and these cavities begin to disappear with the increase in annealing temperature. The surface grain sizes are observed to be smaller in BiVO_4_-400 and BiVO_4_-450, compared to those in BiVO_4_-500 and BiVO_4_-540 samples, which further illustrate the effect of annealing temperature on grain size. The grain size distributions are derived from the SEM images in Fig. 2. Average grain size and grain size range increase with annealing temperature (Table S2†). These results suggest that the higher temperature favors crystals with larger grain size. The estimated coefficients of variation of the grain size indicate that grain size distribution is not uniform at 540 °C (Table S2†). Furthermore, the average grain sizes estimated from SEM images (Fig. 2) show similar trend when compared to the crystal sizes estimated from XRD analyses (Table S3†). These results imply that the BiVO_4_ grains are largely single crystal in each grain in the thin film photoelectrodes prepared especially at lower temperature.

AFM analysis is conducted to investigate surface morphology of the thin film BiVO_4_ photoelectrodes (Fig. S8 and S9†). Root-mean-square roughness (Rms), a reliable parameter for quantifying surface micro-roughness,^45^ is estimated as 12.9 nm, 13.8 nm, 13.7 nm, and 13.5 nm for BiVO_4_-400, BiVO_4_-450, BiVO_4_-500, and BiVO_4_-540, respectively. The annealing temperature shows little effect on surface roughness changes among the BiVO_4_-*n* photoelectrodes.

### 3.4 PEC performances of the photoelectrodes

The PEC performances of BiVO_4_-*n* samples are investigated in a standard three-electrode system and under illumination with a standard AM 1.5G simulated solar light (100 mW cm^−2^). The irradiated was incident on the backside of the BiVO_4_ photoelectrodes, *i.e.*, on the side with no exposed BiVO_4_ thin film. The potential is reported against the reversible hydrogen electrode (RHE) with the following equation:2
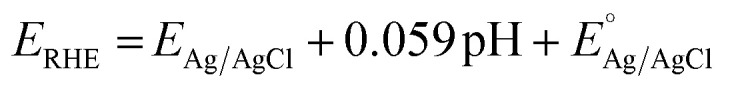
where, *E*_RHE_ is the potential of working electrode against the electrolyte solution with respect to RHE, *E*_Ag/AgCl_ is the potential of working electrode measured with respect to silver chloride electrode, pH is that of the electrolyte solution and 
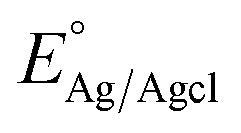
 is the potential of the silver chloride electrode with respect to normal hydrogen electrode (NHE), which is 0.1976 V at 25 °C.

**Fig. 3:**
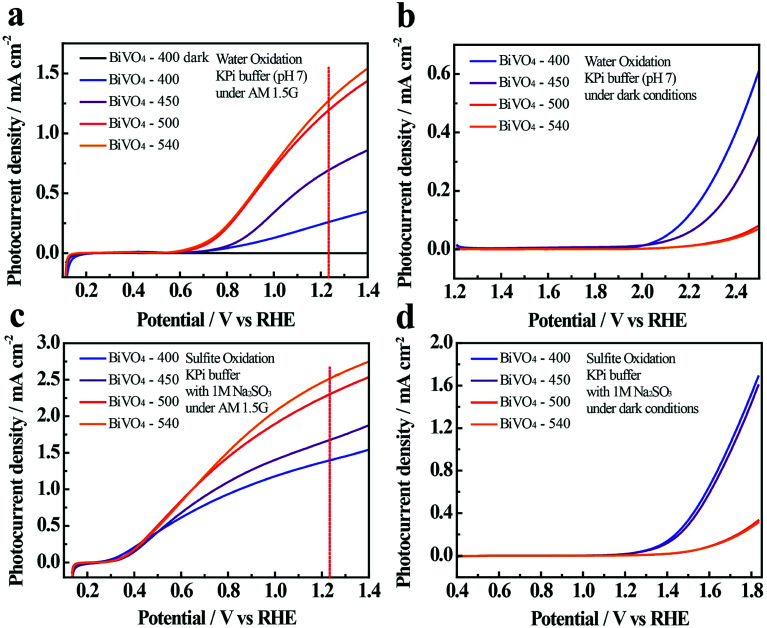
Linear sweep voltammetry (LSV) curves of BiVO_4_-*n* samples obtained at a scan rate of 5 mV s^−1^ in dark or under AM 1.5 illumination. Water oxidation in 0.1 M potassium phosphate (KPi) buffer (pH-7) under illuminated (a) and dark (b) conditions. Sulfite oxidation in 0.1 M KPi, containing 1 M Na_2_SO_3_ under illuminated (c) and dark (d) conditions.

The linear sweep voltammetry (LSV) curves presented in Fig. 3a show that the photocurrents increase with increasing potential, which is expected when enhanced band bending occurs, and when electron–hole recombination (at high potential) is reduced.^46^ The photocurrents at 1.23 V *vs.* RHE in 0.1 M KPi solution increase with increasing annealing temperature (0.25, 0.69, 1.18, and 1.27 mA cm^−2^ for BiVO_4_-400, BiVO_4_-450, BiVO_4_-500 and BiVO_4_-540, respectively) (Fig. 3a).

To investigate the surface reaction kinetics of BiVO_4_-*n* samples, the PEC performances of the BiVO_4_ photoelectrodes are also measured in the presence of a hole-scavenger in the electrolyte (*i.e.*, in this case, 1 M Na_2_SO_3_ in 0.1 M KPi) (Fig. 3c and d). Results indicate that PEC performance is enhanced with sulfite oxidation (Fig. 3c) compared to water oxidation (Fig. 3a). The photocurrent densities are 1.39, 1.68, 2.31, and 2.53 mA cm^−2^ for BiVO_4_-400, BiVO_4_-450, BiVO_4_-500 and BiVO_4_-540, respectively at 1.23 V *vs.* RHE.

The comparison provides convincing evidence of poor water oxidation kinetics by the BiVO_4_ thin film photoelectrodes, which is likely caused by the competition between the surface charge transfer and charge recombination.^47^

The poor water oxidation kinetics of BiVO_4_-*n* samples is also evidenced in the large photocurrent transients, when illumination is turned on and off (Fig. S10†). The photoresponse of the samples over time is measured at 1.23 V *vs.* RHE in 0.1 M KPi buffer (Fig. S10a†) and in 0.1 M KPi containing 1 M Na_2_SO_3_ (Fig. S10b†) under AM 1.5G illumination with 50 s light on/off cycles. Normalized photocurrent transient behaviors are also depicted in Fig. S10.† For water oxidation, an anodic photocurrent spike appears for all samples as soon as the light is turned on, and it decreases thereafter; which is supposedly caused by charge recombination and slow interfacial hole transfer kinetics of BiVO_4_ during water oxidation.^47,48^ In contrast, the presence of the hole scavenger (*i.e.*, Na_2_SO_3_) eliminates the sharp photocurrent spike for all samples, which is attributed to depressed charge recombination due to the facilitated hole transfer kinetics.^9,49,50^ Surprisingly, when in dark, BiVO_4_-400 and BiVO_4_-450 show a cathodic shift of the onset potential relative to BiVO_4_-500 and BiVO_4_-540 and exhibit consistently better water oxidation and sulfite oxidation performances (Fig. 3b and d); which implies that BiVO_4_-400 and BiVO_4_-450 have higher surface catalytic-activities.^10^

Electrochemical impedance spectroscopy (EIS) is also performed for studying BiVO_4_-*n* samples' PEC properties. Fig. S11† presents Nyquist plots for BiVO_4_-*n* samples in dark condition and under AM 1.5 irradiation. It is known that the semicircle in a Nyquist plot at high frequencies is characteristic of charge transfer resistance (*R*_ct_). By comparison, it is clear to find that *R*_ct_ of all BiVO_4_-*n* samples under illumination is lower than the *R*_ct_ in dark, which is caused by the increase in electron conductivity of the photoelectrodes under illumination. In dark (Fig. S11a†), BiVO_4_ samples prepared at lower temperature show a smaller *R*_ct_, and such trend is observed to reverse when irradiated (Fig. S11b†). The difference in trend in dark *vs.* irradiated conditions can explain in part, the difference in trend in the PEC performance, as presented earlier.

Incident photon to electron conversion efficiency (IPCE) spectra are measured from 350 nm to 600 nm and calculated using the following equation:^51^

**Fig. 4:**
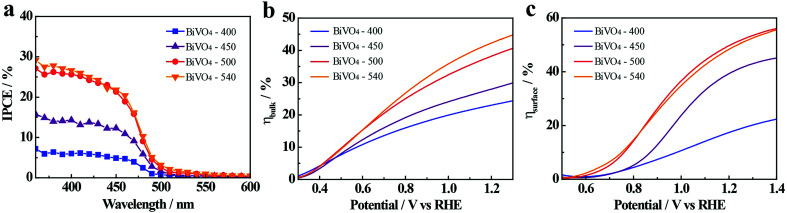
(a) The incident photon to electron conversion efficiency spectra (IPCE) of BiVO_4_-*n* samples, measured at 1.23 V *vs.* RHE in 0.1 M KPi buffer. Charge separation efficiency (b) in the bulk and (c) on the surface.

3 IPCE (%) = [1240*I*(*λ*)/*λJ*_light_(*λ*)] × 100%

where, *λ* is the incident light wavelength, *I*(*λ*) is the measured photocurrent density corresponding to the recorded intensity *J*_light_(*λ*) at the specific wavelength of *λ*. The IPCE values of BiVO_4_-*n* samples for water oxidation increase with annealing temperature at any tested wavelength, with IPCE at 400 nm being 5.7, 14.2, 25.8, and 26.9% for BiVO_4_-400, BiVO_4_-450, BiVO_4_-500 and BiVO_4_-540, respectively (Fig. 4a). The IPCE results are consistent with the previous PEC water oxidation performance, indicating the benefit of high-temperature annealing for BiVO_4_ thin film photoelectrodes.

### 3.5 Electrochemical properties of the photoelectrodes

In order to further explain the effect of annealing temperature on the BiVO_4_ photoelectrodes, the efficiency of charge transport in the bulk (*η*_bulk_) and on the surface (*η*_surface_) of BiVO_4_-*n* samples are determined and presented in Fig. 4b and c (detailed calculation is given in ESI†). The *η*_bulk_ values at 1.23 V *vs.* RHE are 24%, 29%, 35% and 39% for BiVO_4_-400, BiVO_4_-450, BiVO_4_-500 and BiVO_4_-540, respectively, showing an increasing trend with the increase in annealing temperature. Increased bulk separation efficiency with increased annealing temperature is likely due to better crystallinity and increased grain size of BiVO_4_, which decrease recombination defects in the bulk and inside the grains. *η*_surface_ is also improved by high-temperature annealing, with the values at 1.23 V *vs.* RHE being 19%, 41%, 51%, and 50% for BiVO_4_-400, BiVO_4_-450, BiVO_4_-500 and BiVO_4_-540, respectively. Increased surface separation efficiency is likely due to the previously discussed crystal properties that reduce charge recombination centers on the surface of BiVO_4_ thin film photoelectrodes. However, the insignificant difference of *η*_surface_ between BiVO_4_-500 and BiVO_4_-540 deserves further attention.

**Fig. 5:**
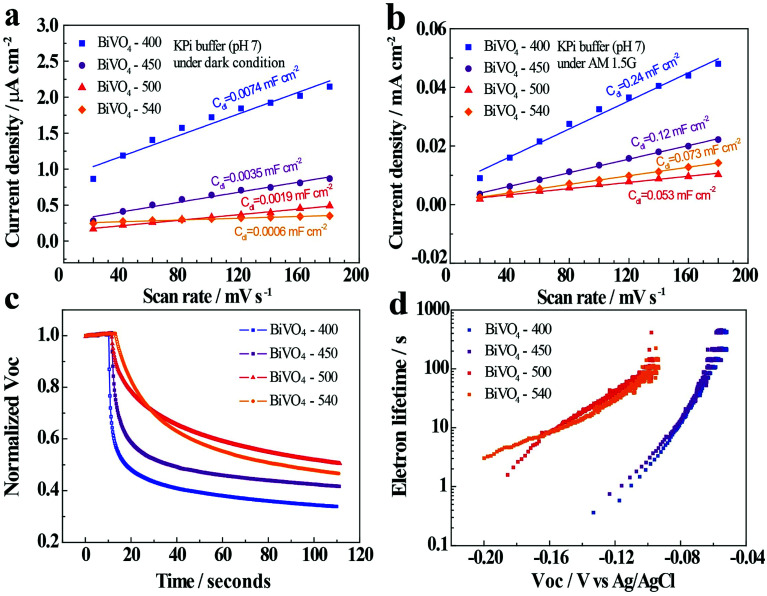
Current density (*J* = 0.5 × (*J*_a_ − *J*_c_)) as a function of scan rates in (a) dark and (b) in AM 1.5G illuminated conditions. *J*_a_ and *J*_c_ present the anodic and cathodic current densities recorded at 0.54 V *vs.* RHE under dark and 0.3 V *vs.* RHE under illuminated conditions. (c) Normalized open-circuit photovoltage decay (*V*_oc_) curves of BiVO_4_-*n* samples in 0.1 M KPi buffer. (d) The potential dependent photoelectron lifetime plots for BiVO_4_-*n*.

Electrochemical double-layer capacitance (*C*_dl_) is positively proportional to the electrochemically active sites. Since it is more convenient to measure *C*_dl_ than quantitatively characterize electrochemically active sites, *C*_dl_ of BiVO_4_-*n* samples are measured to compare the electrochemically active surface areas of these samples. *C*_dl_ is measured through cyclic voltammetry (CV) potentiostatic scans in a non-faradaic potential region with different scan rates ranging from 20 to 180 mV s^−1^ (Fig. S12 and S13†). The potential range is 0.23–0.36 V *vs.* RHE for light condition and 0.47–0.6 V *vs.* RHE for dark. In dark, the *C*_dl_ is determined to be 0.0074, 0.0035, 0.0019, and 0.0006 mF cm^−2^ for BiVO_4_-400, BiVO_4_-450, BiVO_4_-500, and BiVO_4_-540, respectively (Fig. 5a). Therefore, the BiVO_4_ samples prepared at lower temperatures possess higher electrochemically active surface areas compared to the BiVO_4_ samples prepared at higher temperature. The higher electrochemically active surface areas lead to high electrocatalytic activity, which contributes to the cathodic shift of the onset potential under dark condition (Fig. 3d).

Meanwhile, when illuminated, the *C*_dl_ is determined to be 0.240, 0.120, 0.053, 0.073 mF cm^−2^ for BiVO_4_-400, BiVO_4_-450, BiVO_4_-500, and BiVO_4_-540, respectively (Fig. 5b). The *C*_dl_ values show a decreasing trend with the increase in the annealing temperature. These results are consistent with that of the dark condition. However, the increased current density in the illuminated condition exhibits an increase in the *C*_dl_ values. In other words, even though the BiVO_4_ samples prepared at lower temperatures produce higher electrochemically active surface areas, their photocurrents are lower than the BiVO_4_ samples prepared at higher temperature (Fig. 3).

In order to better assess the contrasting effects of annealing temperatures on EC in dark and PEC performance under light illumination, open-circuit photovoltage decay (OCPVD) is measured for all the BiVO_4_-*n* samples (Fig. S14†). OCPVD consists of turning off the illumination at a steady state and monitoring the subsequent decay of open-circuit photovoltage (*V*_oc_) with time. In open-circuit condition under illumination, photo-excitation causes gradual accumulation of electrons in BiVO_4_ photoelectrode, which results in an upshift of the quasi-Fermi level and built-up of *V*_oc_.^52,53^ Since no current can flow to the external circuit, the electron accumulation is in competition against the dissipation processes, particularly, against charge recombination.^54^ When illumination is turned off, the charge recombination process solely dominates, leading to *V*_oc_ decay. It indicates that a longer *V*_oc_ decay time points to a smaller charge recombination rate of the sample. Furthermore, the normalized *V*_oc_ after the light is turned off for all the BiVO_4_ samples are presented in Fig. 5c. The OCPVD result shows that the BiVO_4_ thin film photoelectrode annealed at higher temperature has the longer *V*_oc_ decay time, suggesting its less charge recombination. The *V*_oc_ decay rate is directly related to the photoelectron lifetime:4
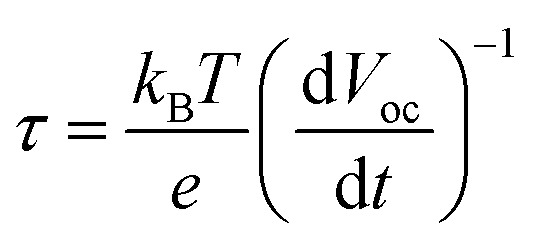
where, *τ* is the potential dependent photoelectron lifetime, *k*_B_ is Boltzmann constant, *T* is the temperature, *e* is elemental electron charge, and *V*_oc_ is the open-circuit voltage at time *t*. The calculated potential dependent photoelectron lifetime is presented in Fig. 5d. It is observed that the photoelectron lifetime increases with decreasing *V*_oc_. The BiVO_4_ samples prepared at higher temperature show relatively longer electron lifetime, leading to their better photocurrent performance.

## 4. Conclusions

The results of this work show that the crystallinity of the BiVO_4_ thin film electrodes is improved and average grain size is increased with the increase in the annealing temperature. A lower annealing temperature produces more electrochemically active sites, which is beneficial to EC performance under dark condition, while a higher annealing temperature leads to less electron–hole recombination and a better PEC performance under illumination. This work provides insights into the effects of annealing temperature in BiVO_4_ thin film photoelectrodes, highlights the importance of controlling annealing temperature of BiVO_4_ for the benefit of enhancing performance, and is thus significant to the future development of metal oxide semiconductor-based thin film photoelectrodes.

## Conflicts of interest

There are no conflicts to declare.

## Supplementary Material

RA-008-C8RA04887H-s001
