# Supplementary material for: Annealing temperature effects on photoelectrochemical performance of bismuth vanadate thin film photoelectrodes
Source: RSC Adv. 2018 Aug 16;8(51):29179–88. doi: 10.1039/c8ra04887h (PMC9084497; doi:10.1039/c8ra04887h)
Supplement: RA-008-C8RA04887H-s001 [file RA-008-C8RA04887H-s001.pdf]

Electronic Supplementary Material (ESI) for RSC Advances.
This journal is © The Royal Society of Chemistry 2018

Supporting Information for

Annealing Temperature Effects on Photoelectrochemical Performance of Bismuth Vanadate Thin Film Photoelectrodes

Le Shi[a], Sifei Zhuo[a], Mutalifu Abulikemu[a], Gangaiah Mettela[a], Thangavelu Palaniselvam[a], Shahid Rasul[a], Bo Tang[a], Buyi Yan[b], Navid B. Saleh[c] and Peng Wang[a]*

[a]L. Shi, Dr. S.F. Zhuo, Dr. M. Abulikemu, Dr. G. Mettela, Dr. T. Palaniselvam, Dr. S. Rasul, Dr. B. Tang, Prof. P. Wang

Water Desalination and Reuse Center, Division of Biological and Environmental Sciences and Engineering, King Abdullah University of Science and Technology, Thuwal 23955-6900, Saudi Arabia. E-mail: peng.wang@kaust.edu.sa

[b] B. Y. Yan, Division of Physical Science and Engineering, King Abdullah University of Science and Technology, Thuwal 23955-6900, Saudi Arabia.

[c] Prof. N. B. Saleh, Department of Civil, Architectural and Environmental Engineering, University of Texas at Austin, Austin, TX 78712, USA.

Table S1. Summary of photocurrent densities of pristine $BiVO_4$ photoelectrodes (in the absence of dopant and cocatalyst) prepared by spin-coating method. Note: backside illumination was used unless otherwise noted.

Reference #	Temperature (^oC)			Duration time			Film thickness	Light source	Illuminated area (cm^2)	Photocurrent density (mA/cm^2 at 1.23 V vs. RHE)	Aqueous solution
	dry	Inter-mediate	final	dry (min)	Inter-mediate (min)	final (hour)					
[1]	150	-	550	5	-	1	5 layers	>420nm	-	0.02 at 1.0 V vs. Ag/AgCl	0.5 M Na_2SO_4
[2]	-	500	-	-	30	-	6 layers	AM 1.5G	0.28	0.2	1.0 M Na_2SO_4
[3]	-	500	500	-	10	2	9 layers	AM 1.5G	0.635	0.25	0.1 M KPi, pH 7.0
[4]	RT	500	500	-	10	2	8 layers	AM 1.5G	-	0.3	0.1 M KBi, pH 9.2
[5]	-	450	450	-	20	3	180 nm	AM 1.5G	-	0.76	0.1 M KPi, pH 7.0
[6]	-	350	460	-	10	2	80 nm	AM 1.5G	-	0.12	0.5 M K_2SO_4 pH 5.9
[7]	-	440	470	-	60	5	1 um	AM 1.5G	1.0	0.42	0.5 M Na_2SO_4 pH 6.6
[8]	RT	500	-	-	30	-	6 layers	0.9 Wcm^{-2}	-	0.17	0.5 M Na_2SO_4 pH 5.44
[9]	-	500	500	-	10	8	16 layers	AM 1.5G	-	1.0 at 1.52 V vs. RHE	KPi pH 7
[10]	-	-	500	-	-	2	7 layers	AM 1.5G	Front side	1.0	0.1 M Na_2SO_4
[11]	-	500	500	-	10	2	4 layers	AM 1.5G	-	0.75	0.1M KPi pH 7
[12]	-	450	450	-	30	10	15 layers	AM 1.5G	-	0.86	0.1M KPi pH 6.7
This work	100	500	500	2	5	0.6	6 layers	AM 1.5G	1.0 cm	1.23	0.1M KPi pH 7

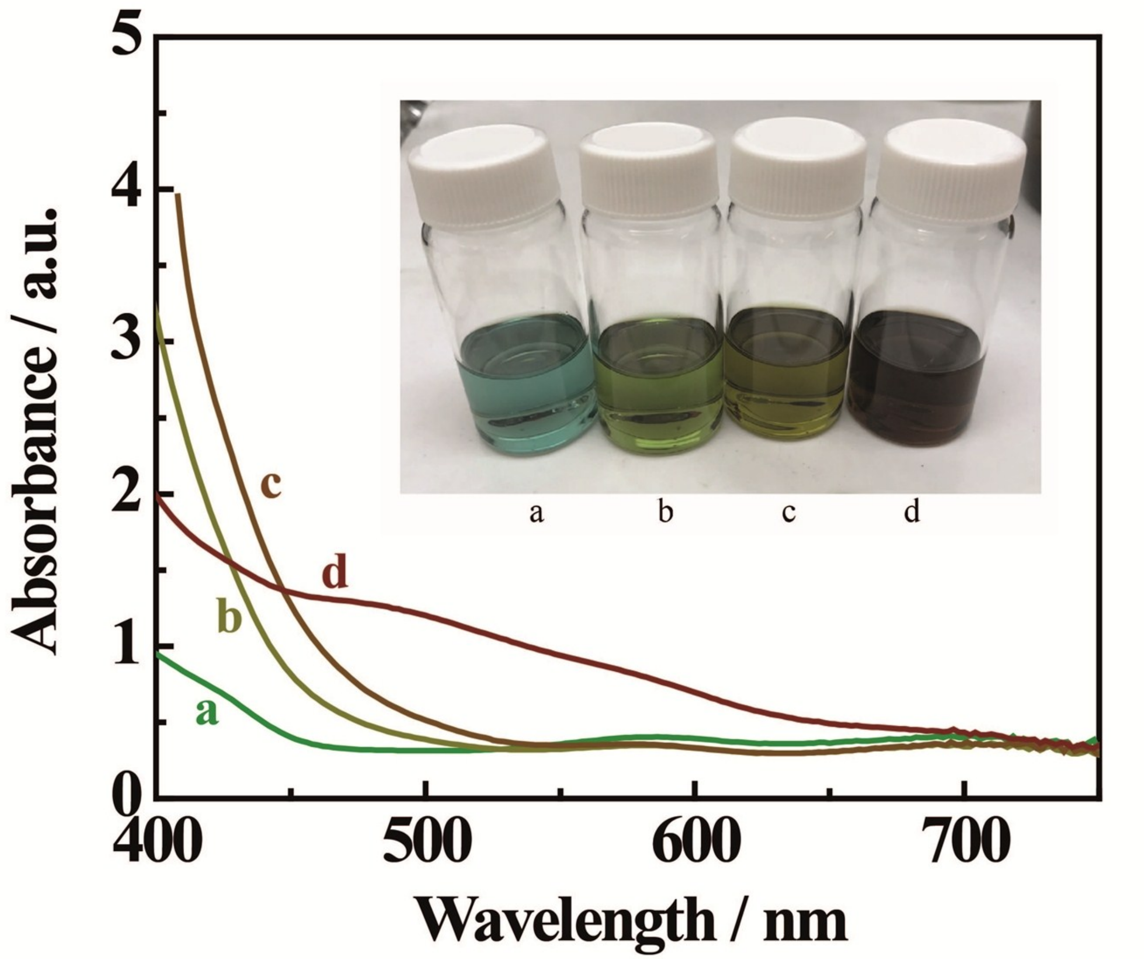

Figure S1. UV-vis absorption spectra of $BiVO_4$ precursor and fresh $VO(acac)_2$ solution. All the solutions are diluted in acetylacetone (1:10). Inset is the photos of (a) fresh $VO(acac)_2$ solution (0.22 g of $VO(acac)_2$ in 10 ml acetylacetone) and $BiVO_4$ precursor (0.4 g of $Bi(NO_3)_3{\cdot}5H_2O$) and 0.22 g of $VO(acac)_2$ in 10 ml acetylacetone): (b) freshly prepared, (c) after continuously stirring for 10 days, (d) sonication with gentle heating for 10 hours.

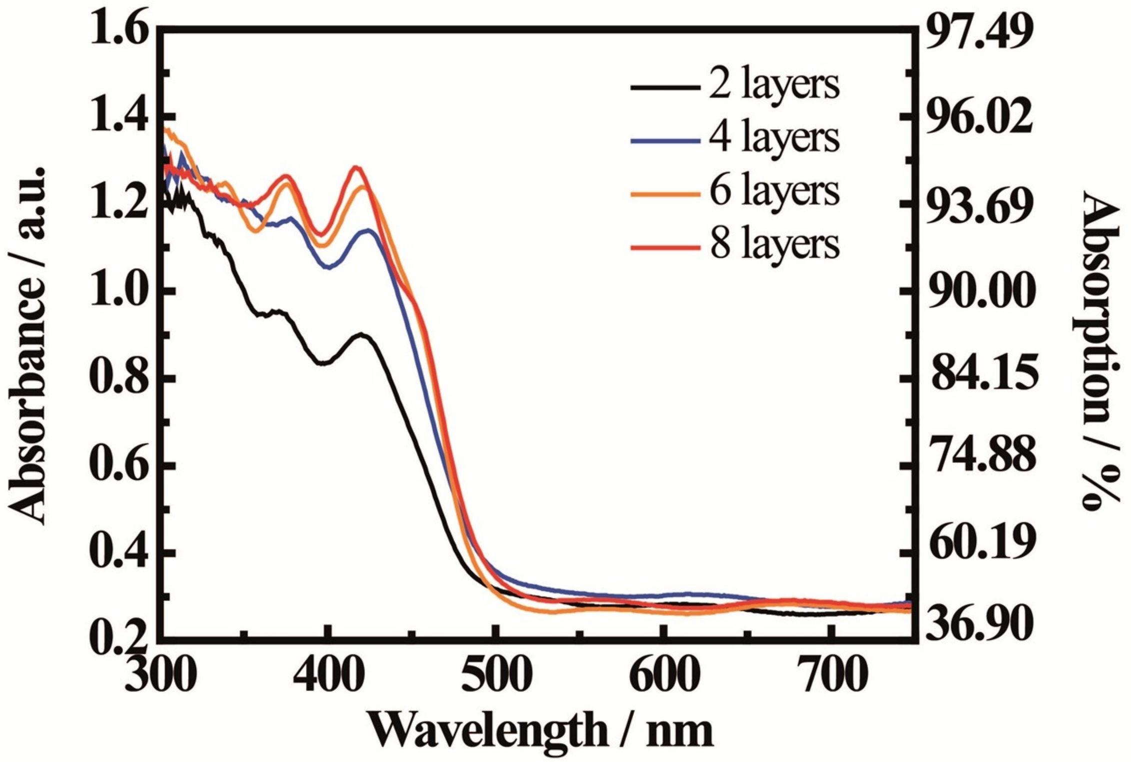

Figure S2. UV-vis absorption spectra of $BiVO_4$ photoelectrode annealed at 500°C with different coating layers.

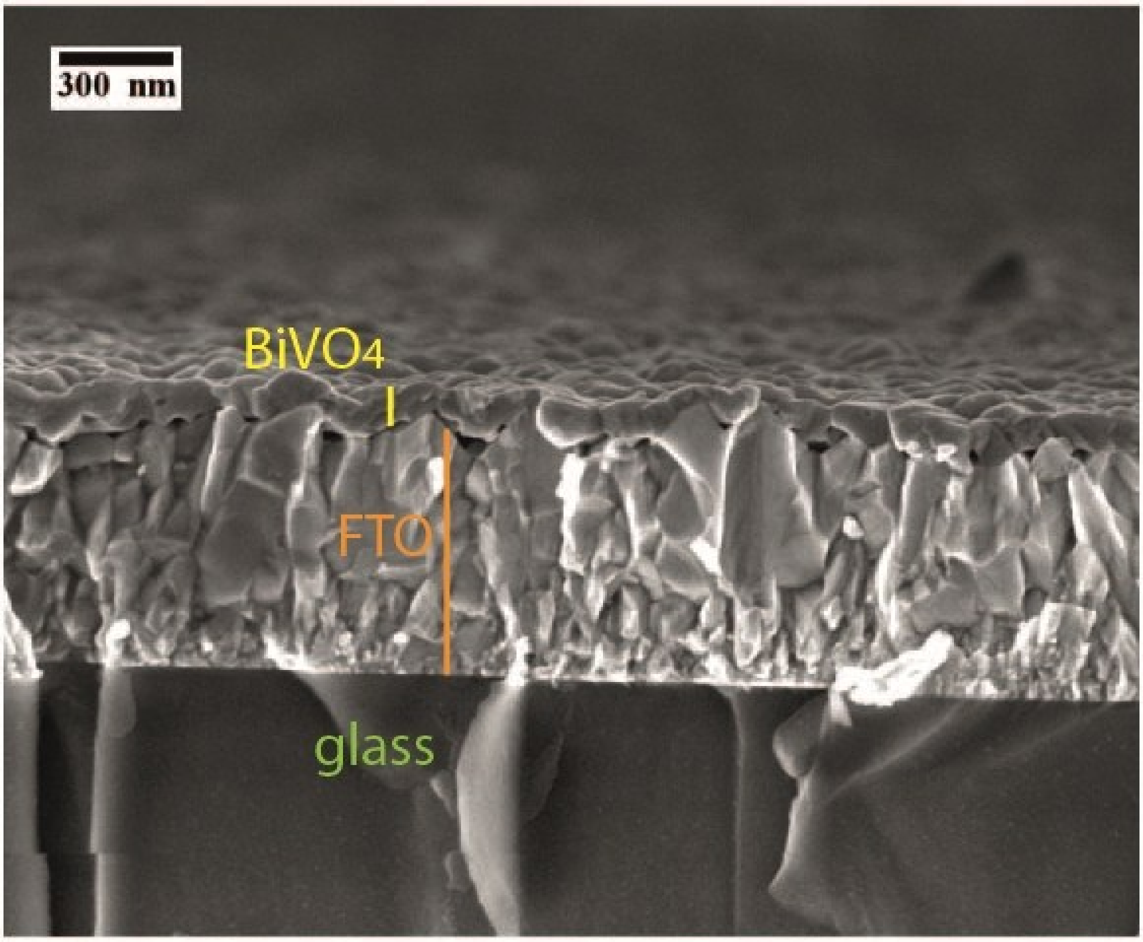

Figure S3. SEM image of cross-section of $BiVO_4$-500. The top layer of $BiVO_4$ thin film is ~93 nm thick.

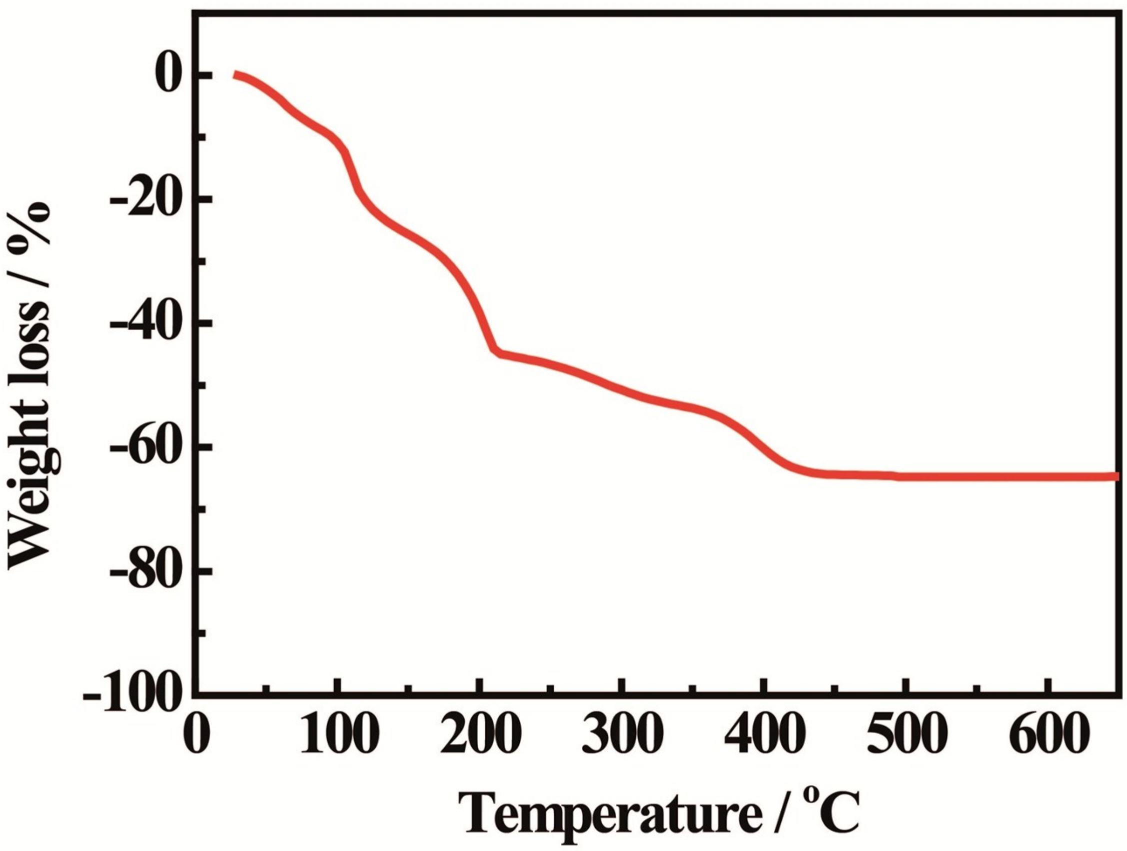

Figure S4. Thermal gravimetric analysis (TGA) of $BiVO_4$ precursor solution. The weight loss was monitored up to 600 ^{o}C with a temperature step of 5 ^{o}C min^{-1}.

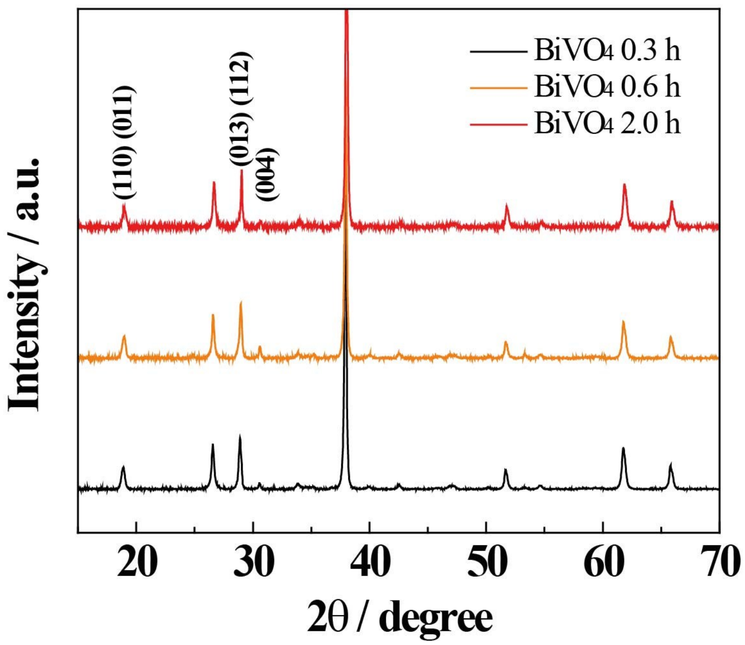

Figure S5. XRD spectra of BiVO-540 with different annealing duration time.

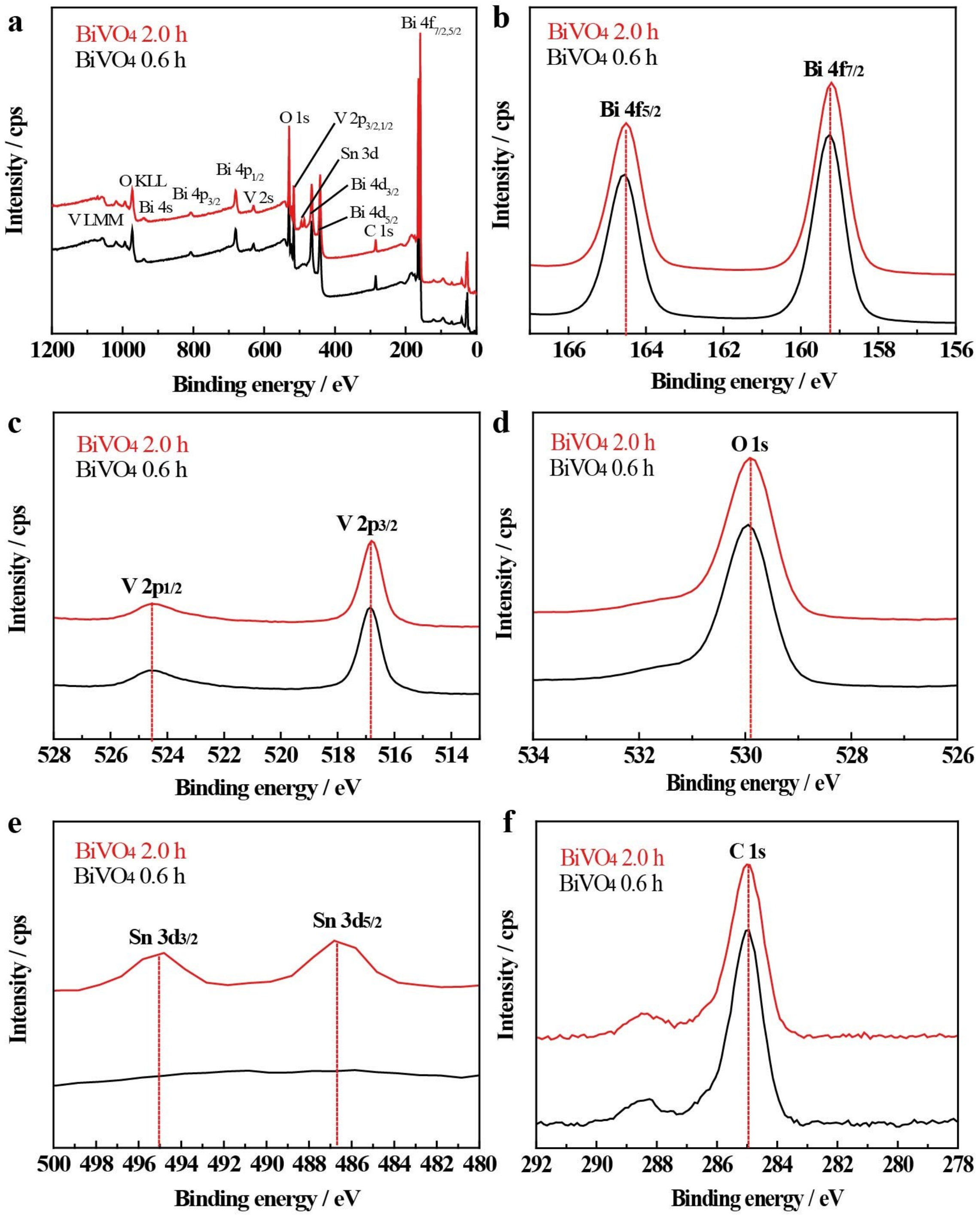

Figure S6. XPS spectra of $BiVO_4$-540 with different annealing duration time: (a) full range survey scan of sample $BiVO_4$-540, (b) individual spectra of Bi 4f, (c) V 2p spectra (d) O 1s spectra, (e) Sn 3d spectra and (f) C 1s spectra.

XPS analysis was carried out to gain addition insights (Figure S6). The wide scan spectra of the sample in Figure S6a indicate the presence of element Bi, V and O. The binding energies of Bi $4f_{7/2}$ (159.2 eV), V $2p_{3/2}$ (516.8 eV) and O 1s (529.9 eV) in $BiVO_4$ are well in accordance with reported $BiVO_4$ literature.[13] The splitting peaks of Bi $4f_{7/2}$ (159.2 eV) and Bi $4f_{5/2}$ (164.5 eV) are shown in Figure S6b, which are characteristics of Bi^{3+}. At the same time, the splitting peaks of V 2p are observed at 524.5 (V $2p_{1/2}$) and 516.8 eV (V $2p_{3/2}$), that occur due to the surface V^{5+} species (shown in Figure S6c). For comparison, $BiVO_4$-500 annealed at 2h is also presented in Figure S6. The additional peaks of Sn $3d_{3/2}$ (495.0 eV) and $Sn3d_{5/2}$ (486.7 eV) are observed, due to the Sn diffusion from FTO substrate during the longer period of annealing. In order to remove the interference from other affects rather than the annealing temperature affect on the $BiVO_4$ material, the 0.6 h annealing duration is chosen based on such Sn diffusion, which has been reported to have uncertain effects on the photocurrent density. [14-17]

Table S2. Grain size information for $BiVO_4$-n photoelectrodes; i.e., range, average grain size, and coefficient of variation.

Samples	Size range (nm)	Average size (nm)	Coefficient of variation
$BiVO_4$-400	10-50	29	0.24
$BiVO_4$-450	10-80	45	0.24
$BiVO_4$-500	20-220	107	0.31
$BiVO_4$-540	30-360	138	0.50

Table S3. XRD crystallite size calculation based on (013) (112) peaks.

**Peak position**	**Annealing Temperature**	**Peak position**	**(FWHM) Peak Width**	**Crystallite size**
(013) (112)	400°C	28.91	0.41	25
	450°C	28.93	0.31	35
	500°C	28.94	0.23	52
	540°C	28.96	0.22	53

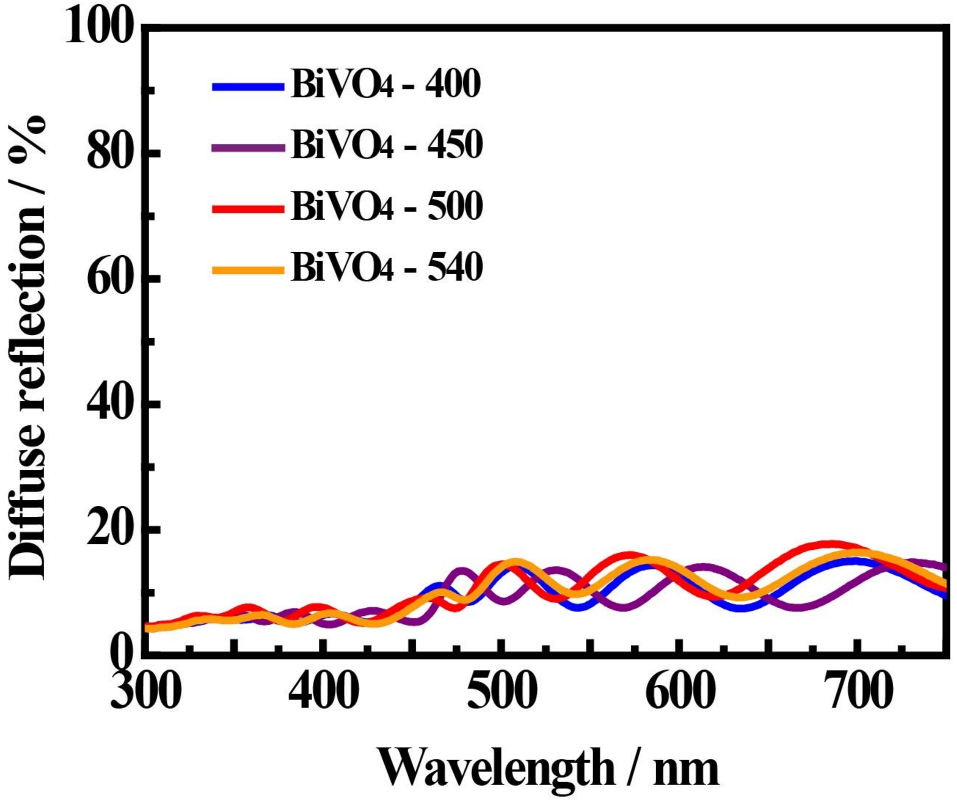

Figure S7. UV-Vis diffuse reflection spectra $BiVO_4$-400, $BiVO_4$-450, $BiVO_4$-500 and $BiVO_4$-540 photoelectrodes with 6 coating layers.

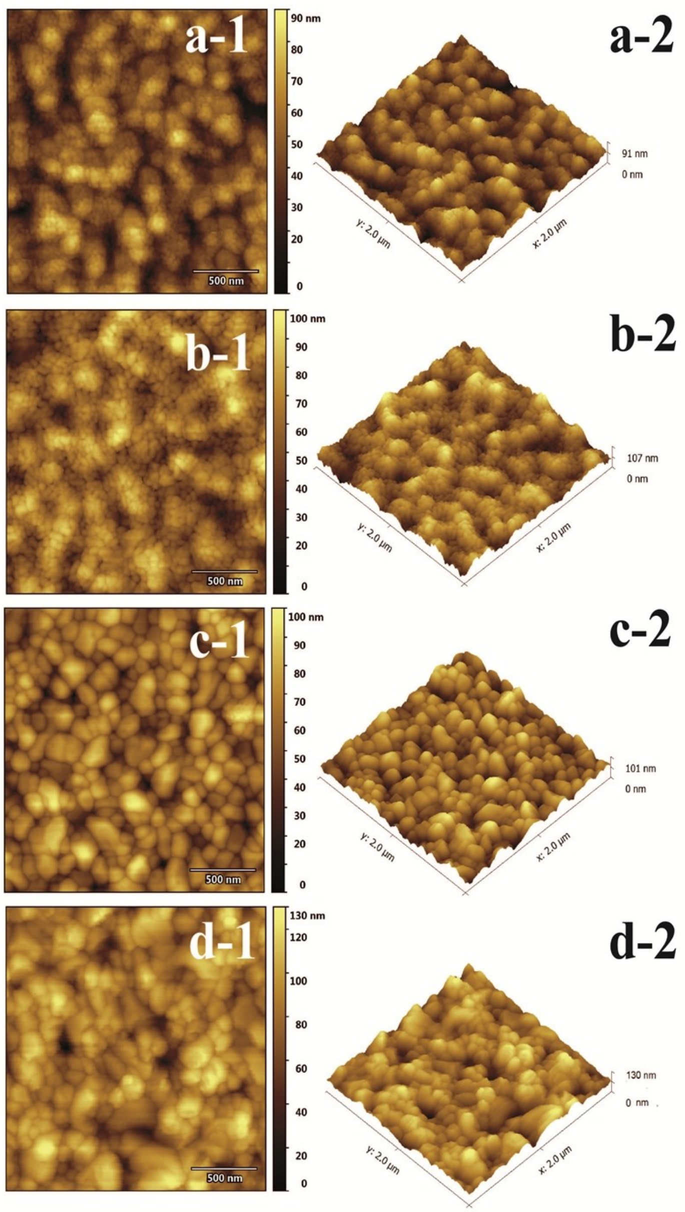

Figure S8. Topographic AFM images of (a1-a2) $BiVO_4$-400 (Rms = 12.9 nm), (b1-b2) $BiVO_4$-450 (Rms = 13.8 nm), (c1-c2) $BiVO_4$-500 (Rms = 13.8 nm), and (d1-d2) $BiVO_4$-540 (Rms = 13.5 nm). The scanned areas was 2.0 × 2.0 µm in all cases.

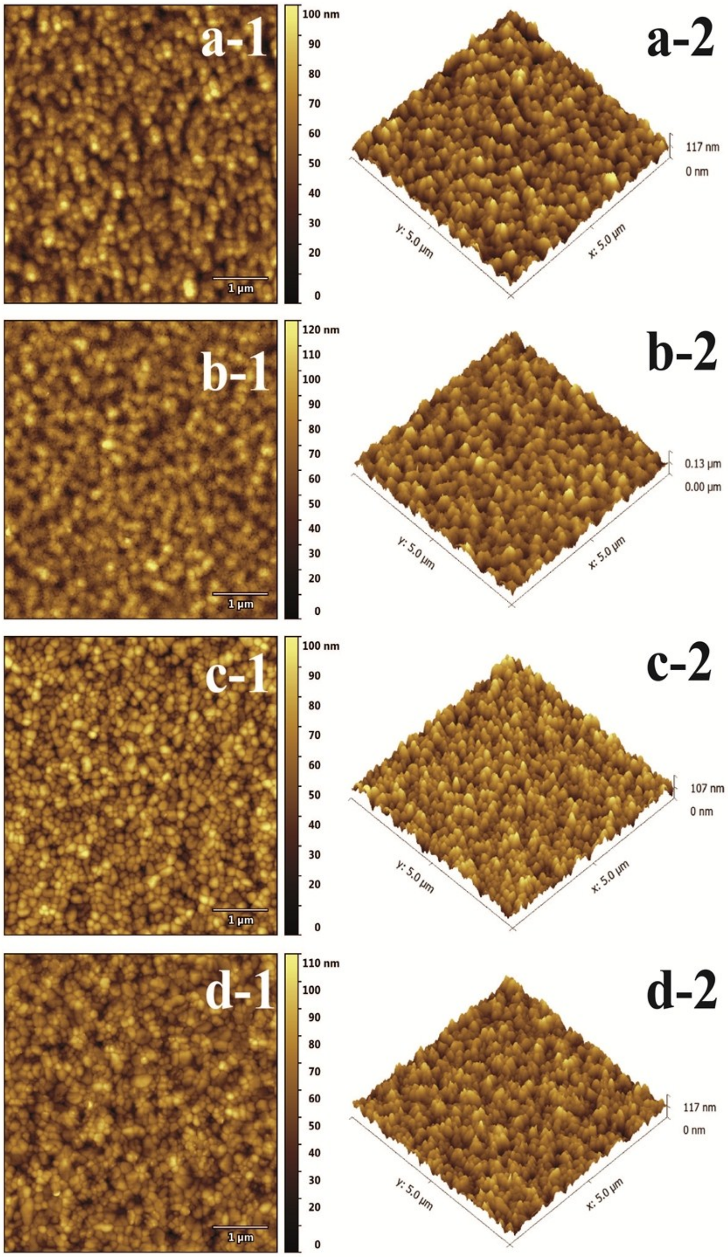

Figure S9. Topographic AFM images of the sample (a1-a2) $BiVO_4$-400 (Rms = 13.8 nm), (b1-b2) $BiVO_4$-450 (Rms = 14.5 nm), (c1-c2) $BiVO_4$-500 (Rms = 13.7 nm) and (d1-d2) $BiVO_4$-540 (Rms = 13.6 nm). The scanned area was 5.0 × 5.0 μm in all.

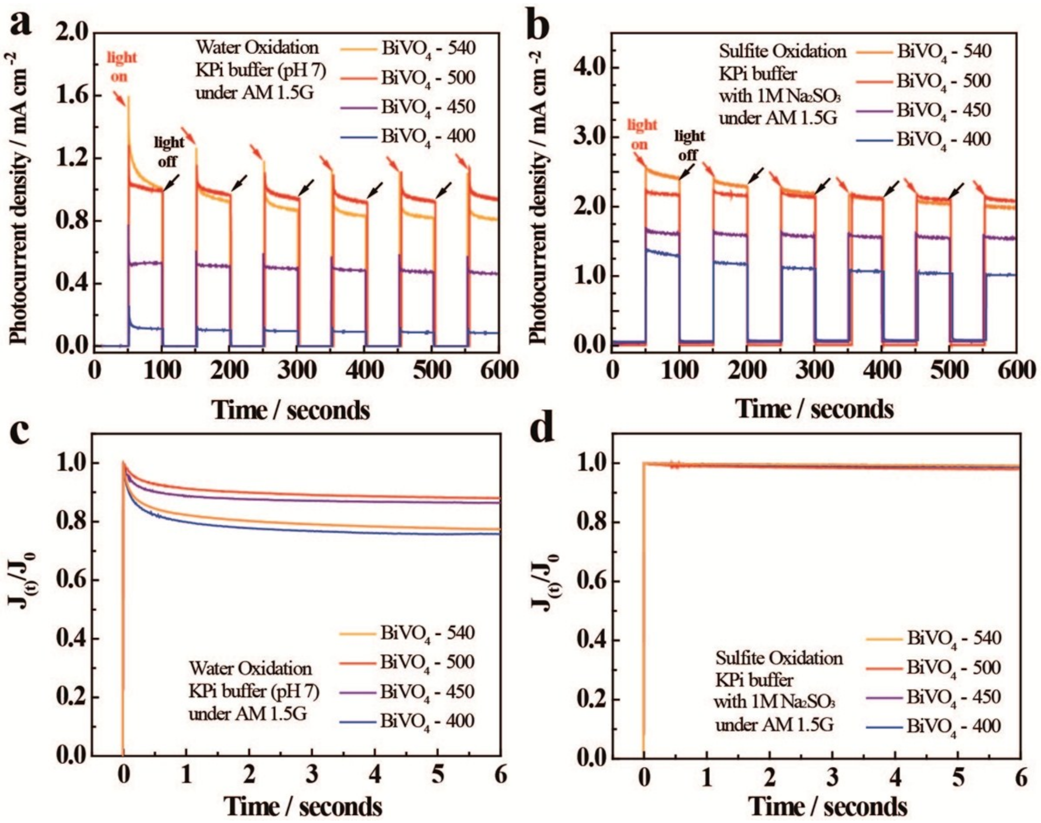

Figure S10. Amperometric I-t curves of $BiVO_4$-n samples, measured at 1.23 V vs RHE in 0.1 M KPi buffer (a) and 0.1 M KPi containing 1 M Na_2SO_3 (b) under AM 1.5G illumination condition with a 50 s light on/off cycles. (c) and (d) are normalized photocurrent transient behaviors of $BiVO_4$-n samples, based on the fourth cycle in (a) and (b) respectively. J_0 is the photocurrent peak measured when t equals to 0.

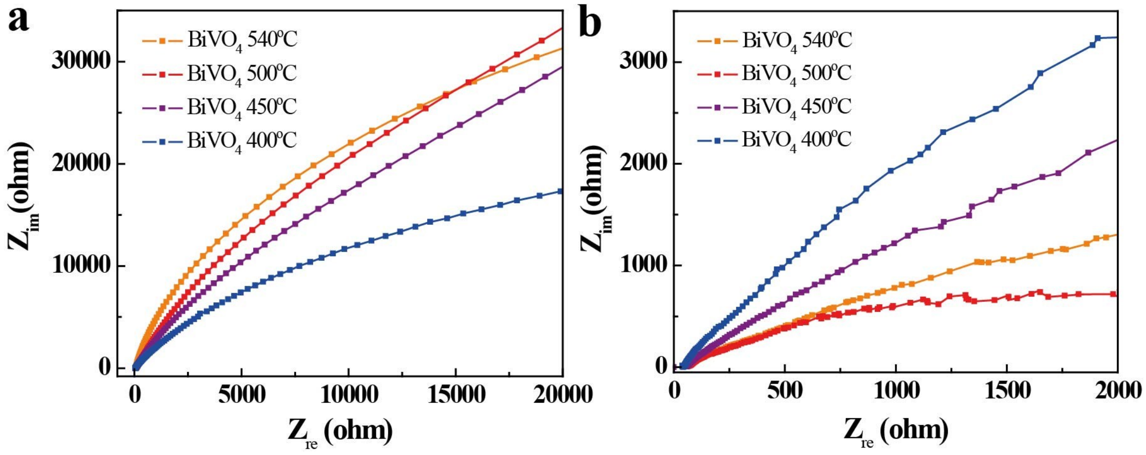

Figure S11 Nyquist plots (Z_{re} vs. Z_{im}) for (a) $BiVO_4$-n samples under dark condition and (b) $BiVO_4$-n samples under AM 1.5 illumination in 0.1M KPi buffer.

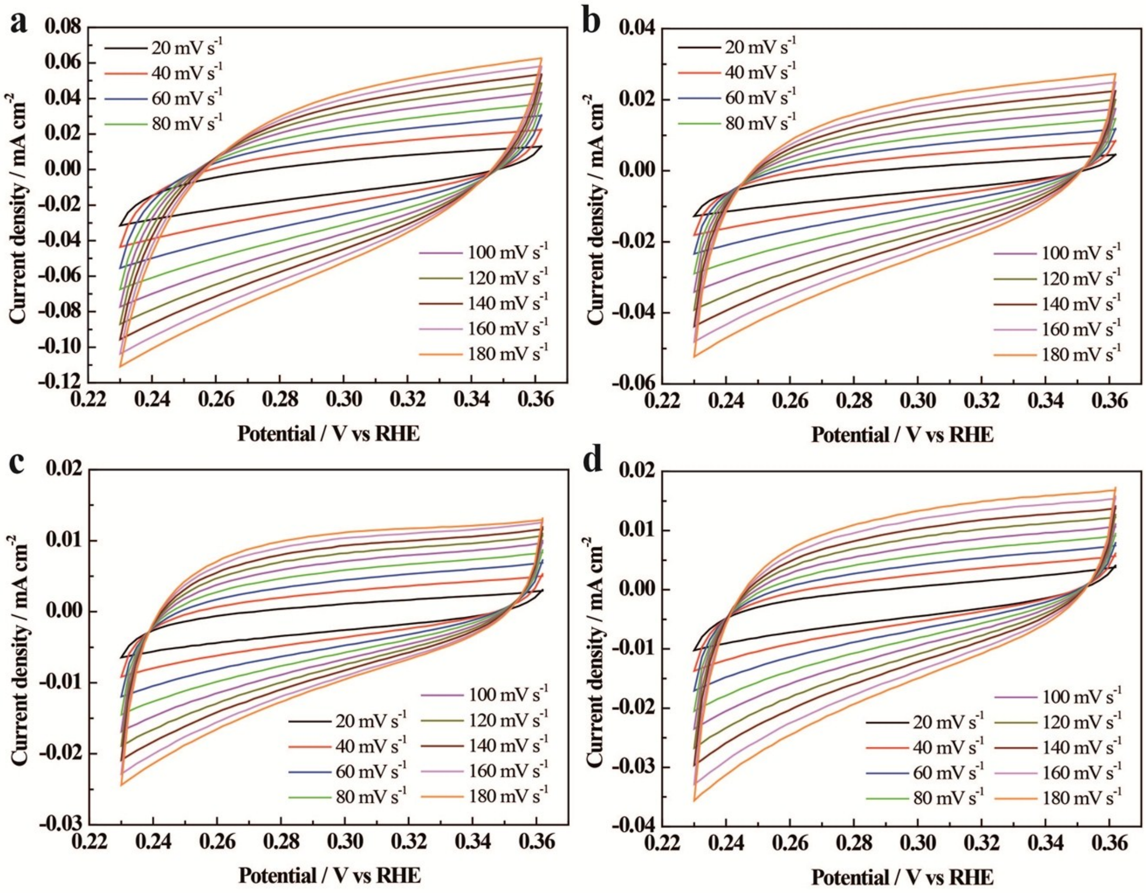

Figure S12 Cyclic voltammetry (CV) potentiostatic curves of $BiVO_4$-400 (a), $BiVO_4$-450 (b), $BiVO_4$-500 (c) and $BiVO_4$-540 (d) measured under light condition, in 0.1 M KPi buffer in the non-Faradaic region with different scan rates from 20 mV s^{-1} to 180 mV s^{-1}.

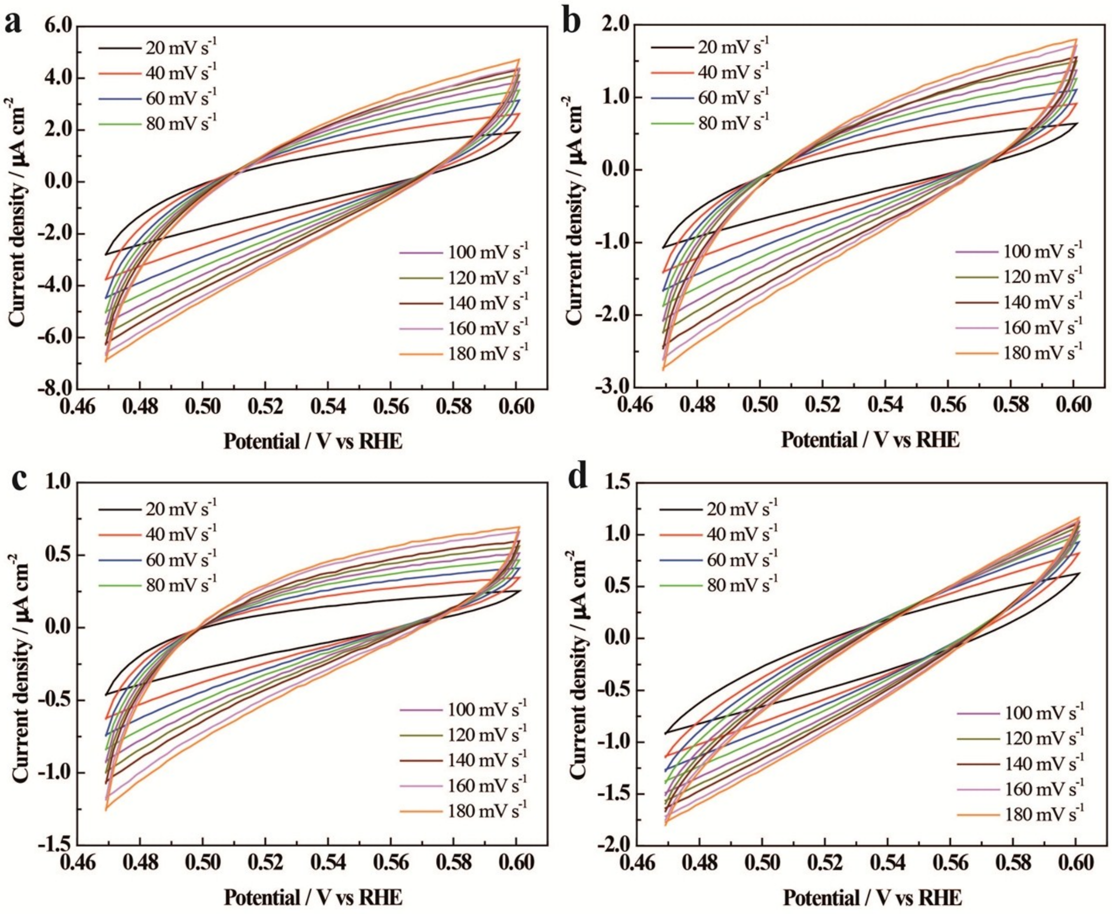

Figure S13 CV curves of $BiVO_4$-400 (a), $BiVO_4$-450 (b), $BiVO_4$-500 (c) and $BiVO_4$-540 (d) measured under dark condition, in 0.1 M KPi buffer in the non-Faradaic region with different scan rates from 20 mV s^{-1} to 180 mV s^{-1}.

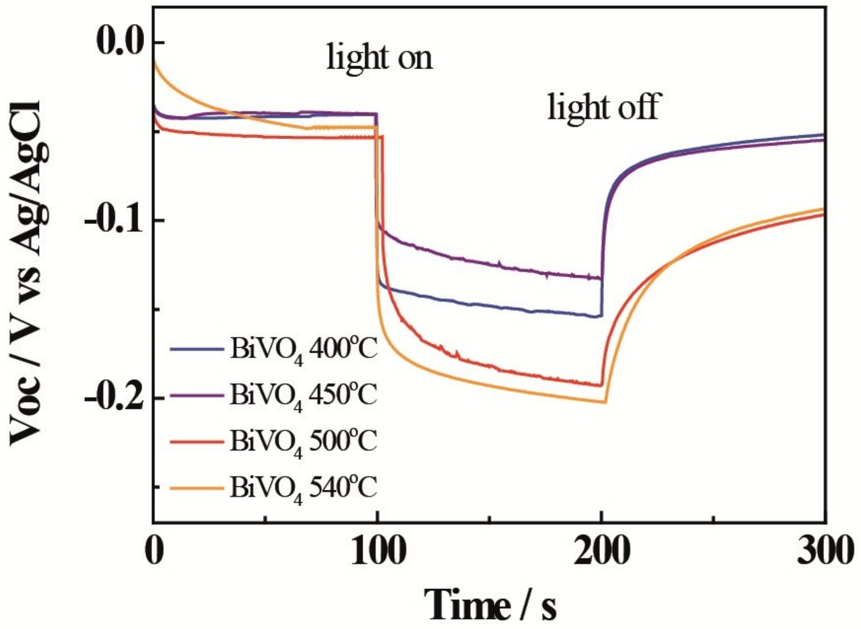

Figure S14 Time dependence of Voc curves measured in 0.1 M KPi buffer in the dark and under AM 1.5G illumination.

Calculation of bulk and surface charge separation efficiency

The photocurrent density (J_{PEC}, which can be retrieved from LSV) of a sample is determined by the following equation[18]:

$$J_{PEC} = J_{abs} \times \eta_{bulk} \times \eta_{surface}$$

where J_{abs} is the photocurrent density when absorbed photons into current efficiency (APCE) is 100%, η_{bulk} is the charge separation efficiency in the bulk of sample, and $\eta_{surface}$ is the charge separation efficiency at the surface of the sample.

Electrolyte buffered with 0.1 M KPi containing 1 M Na_2SO_3 is reasonably assumed to completely suppress the surface recombination of charge carriers without influencing the charge separation in bulk of the sample electrode, which means $\eta_{surface}$ is 100% in the case with 1 M Na_2SO_3 in the electrolyte.

Therefore, η_{bulk} and $\eta_{surface}$ can be determined as follows:

$$\eta_{bulk} = J_{sulfite} / J_{abs}$$

$$\eta_{surface} = J_{water} / J_{sulfite}$$

where J_{water} and $J_{sulfite}$ are the photocurrent density for PEC water oxidation and sulfite oxidation, respectively. By overlapping light absorption efficiency (Figure 4a in the manuscript) and AM 1.5G spectrum, the J_{abs} of $BiVO_4$-n is calculated to be 6.03 mA cm^{-2} for $BiVO_4$-400, 5.94 $mAcm^{-2}$ for $BiVO_4$-450, 5.98 $mAcm^{-2}$ for $BiVO_4$-500 and 5.91 mA cm^{-2} for $BiVO_4$-540. As a result, η_{bulk} and $\eta_{surface}$ of $BiVO_4$-n are calculated and presented in Figure 7b and 7c.

Reference

1. Chatchai, P.; Murakami, Y.; Kishioka, S.-y.; Nosaka, A. Y.; Nosaka, Y., Efficient photocatalytic activity of water oxidation over WO3/BiVO4 composite under visible light irradiation. *Electrochimica Acta* **2009,** *54* (3), 1147-1152.
2. Kazuhiro, S.; Nini, W.; Yugo, M.; Hitoshi, K.; Nobuko, O.-K.; Hideki, S., Effect of Carbonate Ions on the Photooxidation of Water over Porous BiVO4 Film Photoelectrode under Visible Light. *Chemistry Letters* **2010,** *39* (1), 17-19.
3. Jeong, H. W.; Jeon, T. H.; Jang, J. S.; Choi, W.; Park, H., Strategic Modification of BiVO4 for Improving Photoelectrochemical Water Oxidation Performance. *The Journal of Physical Chemistry C* **2013,** *117* (18), 9104-9112.
4. Choi, S. K.; Choi, W.; Park, H., Solar water oxidation using nickel-borate coupled BiVO4 photoelectrodes. *Physical Chemistry Chemical Physics* **2013,** *15* (17), 6499-6507.
5. Xia, L.; Bai, J.; Li, J.; Zeng, Q.; Li, L.; Zhou, B., High-performance BiVO4 photoanodes cocatalyzed with an ultrathin α-Fe2O3 layer for photoelectrochemical application. *Applied Catalysis B: Environmental* **2017,** *204* (Supplement C), 127-133.
6. Chemseddine, A.; Ullrich, K.; Mete, T.; Abdi, F. F.; van de Krol, R., Solution-processed multilayered BiVO4 photoanodes: influence of intermediate heat treatments on the photoactivity. *Journal of Materials Chemistry A* **2016,** *4* (5), 1723-1728.
7. Parmar, K. P. S.; Kang, H. J.; Bist, A.; Dua, P.; Jang, J. S.; Lee, J. S., Photocatalytic and Photoelectrochemical Water Oxidation over Metal-Doped Monoclinic BiVO4 Photoanodes. *ChemSusChem* **2012,** *5* (10), 1926-1934.
8. Quiñonero, J.; Lana–Villarreal, T.; Gómez, R., Improving the photoactivity of bismuth vanadate thin film photoanodes through doping and surface modification strategies. *Applied Catalysis B: Environmental* **2016,** *194* (Supplement C), 141-149.
9. Li, T.; He, J.; Peña, B.; Berlinguette, C. P., Curing BiVO4 Photoanodes with Ultraviolet Light Enhances Photoelectrocatalysis. *Angewandte Chemie International Edition* **2016,** *55* (5), 1769-1772.
10. Kalanur, S. S.; Yoo, I.-H.; Park, J.; Seo, H., Insights into the electronic bands of WO3/BiVO4/TiO2, revealing high solar water splitting efficiency. *Journal of Materials Chemistry A* **2017,** *5* (4), 1455-1461.
11. Cheng, B.-Y.; Yang, J.-S.; Cho, H.-W.; Wu, J.-J., Fabrication of an Efficient BiVO4–TiO2 Heterojunction Photoanode for Photoelectrochemical Water Oxidation. *ACS Applied Materials & Interfaces* **2016,** *8* (31), 20032-20039.
12. Ma, Y.; Pendlebury, S. R.; Reynal, A.; Le Formal, F.; Durrant, J. R., Dynamics of photogenerated holes in undoped BiVO4 photoanodes for solar water oxidation. *Chemical Science* **2014,** *5* (8), 2964-2973.
13. Jia, Q.; Iwashina, K.; Kudo, A., Facile fabrication of an efficient BiVO₄ thin film electrode for water splitting under visible light irradiation. *Proceedings of the National Academy of Sciences* **2012,** *109* (29), 11564-11569.
14. Shinde, P. S.; Annamalai, A.; Kim, Ju H.; Choi, Sun H.; Lee, Jae S.; Jang, Jum S., Exploiting the dynamic Sn diffusion from deformation of FTO to boost the

photocurrent performance of hematite photoanodes. *Solar Energy Materials and Solar Cells* **2015,** *141*, 71-79.
15. Yang, Y.; Zhan, F.; Li, H.; Liu, W.; Yu, S., In situ Sn-doped WO3 films with enhanced photoelectrochemical performance for reducing CO2 into formic acid. *Journal of Solid State Electrochemistry* **2017,** *21* (8), 2231-2240.
16. Rachel, M.; Mahfujur, R.; Don, M. J. M.; A., W. C., Activation of Hematite Nanorod Arrays for Photoelectrochemical Water Splitting. *ChemSusChem* **2011,** *4* (4), 474-479.
17. Li, M.; Deng, J.; Pu, A.; Zhang, P.; Zhang, H.; Gao, J.; Hao, Y.; Zhong, J.; Sun, X., Hydrogen-treated hematite nanostructures with low onset potential for highly efficient solar water oxidation. *Journal of Materials Chemistry A* **2014,** *2* (19), 6727-6733.
18. Chang, X.; Wang, T.; Zhang, P.; Zhang, J.; Li, A.; Gong, J., Enhanced Surface Reaction Kinetics and Charge Separation of p–n Heterojunction Co3O4/BiVO4 Photoanodes. *Journal of the American Chemical Society* **2015,** *137* (26), 8356-8359.
